# The Impact of Neoadjuvant Chemotherapy on the Surgical Management of Colorectal Peritoneal Metastases: A Systematic Review and Meta-Analysis

**DOI:** 10.1245/s10434-022-11699-7

**Published:** 2022-04-09

**Authors:** Michael P. Flood, Joseph C. H. Kong, Kasmira Wilson, Helen Mohan, Peadar S. Waters, Jacob J. McCormick, Satish K. Warrier, Jeanne Tie, Robert Ramsay, Michael Michael, Alexander G. Heriot

**Affiliations:** 1 Division of Surgical Oncology, Peter MacCallum Cancer Centre, Melbourne, Australia; 2 Sir Peter MacCallum Department of Oncology, University of Melbourne, Melbourne, Australia; 3 Department of Medical Oncology, Peter MacCallum Cancer Centre, Melbourne, Australia

## Abstract

**Background:**

Cytoreductive surgery (CRS) with or without hyperthermic intraperitoneal chemotherapy (HIPEC) is a well-recognised treatment option for the management of colorectal peritoneal metastases (CRPM). However, incorporating the routine use of neoadjuvant chemotherapy (NAC) into this management plan is controversial.

**Methods:**

A systematic review and meta-analysis were conducted to evaluate the impact of neoadjuvant chemotherapy on perioperative morbidity and mortality, and long-term survival of patients with CRPM undergoing CRS and HIPEC.

**Results:**

Twelve studies met the inclusion criteria (*n* = 2,463 patients). Ten were retrospective cohort, one was prospective cohort, and one was a prospective randomised by design. Patients who received NAC followed by CRS and HIPEC experienced no difference in major perioperative morbidity and mortality compared with patients who underwent surgery first (SF). There was no difference in overall survival at 3 years, but at 5 years NAC patients had superior survival (relative risk [RR] 1.31; 95% confidence interval [CI] 1.11–1.54, *P* < 0.001). There were no differences in 1- and 3-year, disease-free survival (DFS) between groups. Study heterogeneity was generally high across all outcome measures.

**Conclusions:**

Patients who received neoadjuvant chemotherapy did not experience any increase in perioperative morbidity or mortality. The potential improvement in 5-year overall survival in patients receiving NAC is based on limited confidence due to several limitations in the data, but not sufficiently enough to curtail its use. The practice of NAC in this setting will remain heterogeneous and guided by retrospective evidence until prospective, randomised data are reported.

**Supplementary Information:**

The online version contains supplementary material available at 10.1245/s10434-022-11699-7.

Peritoneal disease confers the worst prognosis amongst all sites of metastatic colorectal cancer.^1^ The advent of cytoreductive surgery (CRS) and hyperthermic intraperitoneal chemotherapy (HIPEC) now offers a well-recognised treatment option for up to a quarter of highly selected patients with colorectal peritoneal metastases (CRPM), with 5-year survival rates of 30–40%.^2–6^

In a recent landmark, randomised trial (PRODIGE 7) where CRS and oxaliplatin-based HIPEC was compared to CRS alone, 83% of both groups received neoadjuvant chemotherapy (NAC).^7^ The survival data reported for both arms was impressive (41.7 months in the CRS and HIPEC group versus 41.2 months in the CRS group), leading to considerable debate as to the impact of the neoadjuvant, and indeed the adjuvant systemic chemotherapy (AC) in this patient cohort.

The role of neoadjuvant chemotherapy in CRPM is twofold: first to downstage tumour burden while treating micrometastatic systemic disease, but also to aide in patient selection by challenging tumour biology, testing response to chemotherapy, and identifying patients with a favourable disease phenotype.

Current evidence supporting NAC is varied and retrospective in design. Heterogeneity in patient selection, systemic chemotherapy, and intraperitoneal chemotherapy regimens limit interpretation of current data. Two systematic reviews in 2017 highlighted this heterogeneity by not drawing any meaningful conclusions regarding NAC efficacy.^8,9^ In light of this, a multicentre, randomised trial comparing perioperative chemotherapy (neoadjuvant and adjuvant chemotherapy) CRS and HIPEC versus CRS and HIPEC alone is currently recruiting, with completion predicted for June 2026.^10^

In the context of new studies since 2017 and the results of PRODIGE 7, this systematic review and meta-analysis aimed to provide a timely, updated assessment of perioperative and oncological outcomes associated with the application of neoadjuvant systemic chemotherapy in patients with CRPM undergoing CRS and HIPEC.

## Methods

This study protocol was prospectively registered with PROSPERO (registration number: CRD42021274777) and was performed according to the Preferred Reporting Systems for Systematic Reviews and Meta-Analyses (PRISMA).^11^

### Search strategy

A comprehensive search of the literature was undertaken on Ovid MEDLINE, EMBASE, and the Web of Science databases on the August 12, 2021. The following medical subject heading terms (MESH) were used either alone or in combination, using the explode function: colorectal, peritoneum, CRS, HIPEC, neoadjuvant chemotherapy. The search strategy is supplied as Supplementary Data 1. Search results were pooled using the Rayyan online platform (https://rayyan.qcri.org/welcome).

### Inclusion Criteria

Full-text studies with ten or more patients, comparing upfront CRS, HIPEC (surgery first [SF]) to neoadjuvant chemotherapy followed by CRS and HIPEC (NAC) in the treatment of colorectal peritoneal metastases were included. Manual cross-referencing from the bibliographies of papers found in the initial search was undertaken to include additional papers that had not been previously identified. Two reviewers (MF and PW) performed the search and data extraction. The senior authors (AH and MM) independently evaluated any discrepancies in study inclusions or exclusions.

### Exclusion Criteria

Case reports or series of ≤10 patients were excluded. Studies reporting on outcomes from appendiceal or noncolorectal cancers were excluded. Similarly, studies reporting on multiple cancers were screened and excluded if data specific to CRPM could not be extracted. Where articles overlapped or duplicated data, those with the more complete or pertinent material were retained. Conference abstracts were not considered.

### Data Extraction and Analysis

Data for disease-free (DFS) and overall survival (OS) were collected as percentages at selected intervals of follow-up and converted to absolute numbers for pooled analysis. Median survival data was preferentially not meta-analysed, because two studies did not reach this endpoint.^12,13^ Overall survival data were extracted without further stratification by subsequent (adjuvant or palliative) systemic chemotherapy or surgical intervention (iterative CRS & HIPEC or palliation). Categorical data, such as perioperative mortality and major (Clavien-Dindo or CTCAE grade III/IV) morbidity, were similarly collected for the final analysis. The assessment of the safety of NAC could not be addressed in this study due to the lack of reported data on chemotherapy-related toxicities and as to whether these toxicities prevented patients in proceeding to surgery. If any data reported zero events, this was replaced with 0.5 to allow for computation of statistical calculation. A pooled odds ratio (OR) and relative risk (RR) was calculated based on the Cochran-Mantel-Haenszel test and random effect model analysis, respectively. I^2^ statistics was performed to assess for interstudy heterogeneity. *P* < 0.05 was considered significant. All data analysis was performed in R Studio Team 2015 (RStudio: Integrated Development for R Studio, Inc., Boston, MA), using the metaphor package for meta-analysis.

### Risk of Bias Assessment

Two independent reviewers (MF and KW) performed a quality assessment of included studies. This was assessed using the Oxford quality reporting system/Jadad scale for randomised trials^14^ and the Newcastle Ottawa scale for nonrandomised studies^15^ (Tables 1 and 2).

**Table 1:** Newcastle Ottawa Scale for nonrandomized trials

	Author	Year	Selection (4*)	Comparability (2*)	Outcome (3*)	Total
1	Repullo	2021	**	*	***	******
2	Zhou	2021	**	*	**	*****
3	Beal	2020	**		***	*****
4	Leimkuhler	2019	**		***	*****
5	Van Eden	2017	**		***	*****
6	Devilee	2016	**		***	*****
7	Baratti	2014	*		***	****
8	Ceelen	2014	**		**	****
9	Passot	2012	*		***	****
10	Elias	2010	*		***	****
11	Glehen	2004	*		***	****

**Table 2:** Jadad scale for methodological quality

	Author	Year	Randomisation (2)	Blinding (2)	Account of patients (1)	Total
1	Rovers	2021	2	0	1	3

## Results

### Selected Studies

**Fig. 1:**
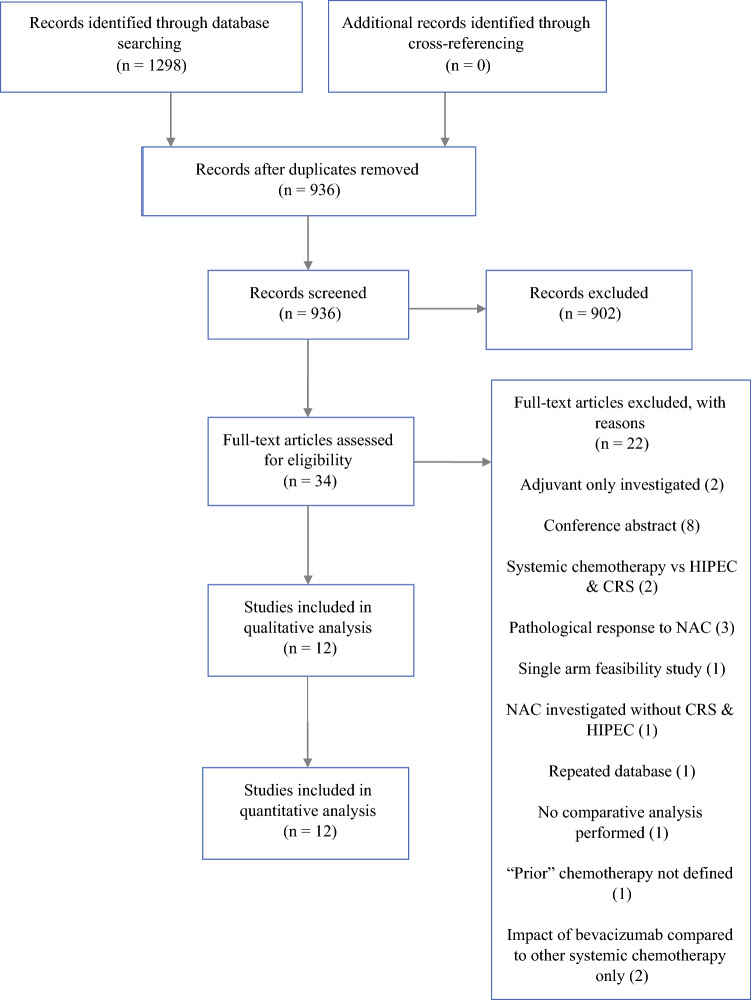
Flow diagram of selected studies

The initial literature search identified 936 articles. After screening, 12 studies were eligible and included in the systematic review and meta-analysis (Fig. 1). Of 2,463 patients included in the 12 studies, 1,273 underwent neoadjuvant systemic chemotherapy followed by CRS and HIPEC (NAC group) and 1,190 underwent upfront CRS and HIPEC (SF group). There was one randomised trial, one prospective cohort, and ten retrospective cohort studies. One study extracted data from the US HIPEC collaborative database.^16^ Five were single-centre experiences, three were national, bi-institutional, and two were international, multicentre. Ten studies originated from Europe, one from the United States, and one from China.

The characteristics of the 12 included studies are listed in Table 3. The number of patients included who underwent NAC in each study ranged from 14 to 370. Two studies only included patients with synchronous CRPM.^12,13^ There were a wide range of NAC regimens, with 5-fluorouracil (5-FU), oxaliplatin, and irinotecan used most frequently. The antivascular endothelial growth factor (VEGF) antibody, bevacizumab, was used (where specifically reported) in 41% (196/480) of patients.^3,12,13,16–19^

**Table 3:** Characteristics of included studies

Author (yr) (study period)	No. of sites (country)	Study design	Primary goal (with respect to patients with CRPM undergoing CRS ± HIPEC)	Onset of Metastases	NAC/SF patients (N)	NAC chemotherapy	Intraperitoneal chemotherapy	Adjuvant chemotherapy (%) NAC/SF	PCI (median) NAC/SF	CC (%) NAC/SF
Zhou (2021) (2017-2019)	2 (China)	Retrospective cohort	Investigate:	Synchronous	20/32	CAPOX	Lobaplatin	80/87.5	9.8/13.7^1^	80/46.9
			Survival benefits &			FOLFIRI	Oxaliplatin			
			Perioperative safety of NAC			FOLFOX	Raltitrexed			
						5-FU	Faltitrexed			
						+/- BEV (30%)				
Repullo (2021) (2008-2017)	2 (Belgium)	Retrospective cohort	Investigate:	Both	56/69	Unknown	Oxaliplatin	Unknown	8/6	100/100
			Disease-free &				Mitomycin			
			Overall survival associated with NAC							
Rovers (2021) (2012-2017)	9 (Netherlands)	RCT	Assess:	Both	37/42	CAPOX	Oxaliplatin	59/0	5/12	89/86
			Feasibility &			FOLFOX	Mitomycin			
			Preoperative & perioperative safety of NAC			FOLFIRI				
						+/- BEV (97%)				
Beal (2020) (2012-2017)	12 (USA)	Retrospective cohort	Assess impact of NAC on:	Both	196/102	FOLFO	Oxaliplatin	40.3/33.3	12.1/14.3^1^	89.8/83.3
			Short-term			FOLFIRI	Mitomycin			
			Long-term outcomes			Capecitabine				
						CAPOX				
						5-FU				
						+/- BEV (54.1%)				
Leimkuhler (2019) (2013-2015)	1 (Germany)	Prospective cohort	Assess:	Both	14/88	CAPOX	Mitomycin	Unknown	15 (NAC)	66 (NAC)
			Feasibility &							
			Perioperative safety of NAC							
Van Eden (2017) (2004-2015)	1 (Netherlands)	Retrospective cohort	Evaluate effect of timing of chemotherapy on survival	Both	78/202	CAPOX	Mitomycin	33/68	Unknown	94.9/90.5
						FOLFOX	Oxaliplatin			
Devilee (2016) (2007-2014)	1 (Netherlands)	Retrospective cohort	Compare:	Synchronous	25/66	CAPOX	Mitomycin	84/89	6/8	96/97
			Short-term &			Capecitabine				
			Long-term outcomes of NAC			FOLF OX				
						+/- BEV (28%)				
Baratti (2014) (2004-2012)	2 (Italy)	Retrospective cohort	Impact of major post-operative complications on oncological outcomes	Both	51/50	CAPOX	Cisplatin	69 (Entire group)	10 (Entire group)	87 (Entire group)
						FOLFOX	Mitomycin			
						FOLFIRI				
						5-FU				
						+/- BEV/CET (31%)				
Ceelen (2014) (2002-2012)	1 (Belgium)	Retrospective cohort	Assess benefit of NAC with the addition of biologic therapy	Both	61/105	FOLFOX	Oxaliplatin	50 (Entire group)	Unknown	87.3 (Entire group)
						FOLFIRI	Mitomycin			
						+/- BEV (16%)				
Passot (2012) (1991-2010)	1 (France)	Retrospective cohort	Prognostic impact of NAC	Both	90/30	FOLFOX	Mitomycin	63 (Entire group)	8.2^1^ (Entire group)	86.1 (Entire group)
						FOLFIRI	Irinotecan			
						FOLFOXIRI	Oxaliplatin			
						+/- BEV/CET (19%)				
Elias (2010) (1990-2007)	23 (International)	Retrospective cohort	Assess:	Both	370/173	Unknown	Mitomycin	47 (Entire group)	Unknown	95 (Entire group)
			Early &				Oxaliplatin			
			Long-term survival of patients treated with CRS & HIPEC							
Glehen (2004) (1987-2002)	28 (International)	Retrospective cohort	To evaluate the efficacy of CRS & HIPEC	Both	275/231	FOLFOX	Mitomycin	40.3 (Entire group)	Unknown	74.3 (Entire group)
						FOLFOXIRI	Cisplatin			
						5-FU	Oxaliplatin			
						Cisplatin+5-FU	5-FU (EPIC)			

*NAC* neoadjuvant chemotherapy; *SF* surgery first; *PCI* peritoneal carcinomatosis index; 5-FU, 5-fluorouracil; FOLFOX, 5-FU, oxaliplatin, leucovorin; FOLFIRI, 5-FU, irinotecan, leucovorin; *BEV* bevacizumab; *CET* cetuximab; FOLFIRINOX/FOLFOXIRI, 5-FU, oxaliplatin, irinotecan, leucovorin; *CAPOX* capecitabine, oxaliplatin; *NR* not reached; *NA* not available; *CC* complete cytoreduction (CC0/CC1).

^1^Mean value

Both the mode and regimen of intraperitoneal chemotherapy varied substantially. Oxaliplatin, mitomycin C, cisplatin, irinotecan, lobaplatin, raltitrexed, and faltitrexed were used in combination or as sole agents as HIPEC. 5-FU was used as early postoperative intraperitoneal chemotherapy (EPIC) in one study.^20^ Five studies reported on adjuvant systemic chemotherapy by group (NAC versus SF).^12,13,16,19,21^ Of these, 46% (164/356) of patients who received NAC also received adjuvant chemotherapy compared with 58% (258/444) of patients who underwent surgery first. Six studies reported on mean/median PCI^12,13,16,19,22,23^ and seven studies reported on the completeness of cytoreduction.^12,13,16,19,21–23^ Operative mean/median PCI ranged from 5 to 15 in the NAC group and 6–14.3 in the SF group. Complete cytoreduction (CC0/1) ranged from 66% to 100% in the NAC group and 46.9–100% in the SF group. Survival data for patients who underwent CRS and HIPEC with or without NAC are listed in Table 4.

**Table 4:** Survival data from included studies

Author (year)	Median follow-up (mo)	OS (%)			DFS (%)			Overall mortality (%)	Grade III/IV morbidity (%)	Median DFS (mo)	Median OS (mo)
		1-year	3-year	5-year	1-year	3-year	5-year				
Zhou (2021)	18.5	NAC = 79*	NAC = 67.4	–	–	–	–	NAC = 0	NAC = 40	–	NAC = NR*
		SF = 55	SF = 32.2 (2-year)					SF = 0	SF = 31.3		SF = 20
Repullo (2021)	54	NAC = 98	NAC = 59	NAC = 35	NAC = 47	NAC = 13	NAC = 6	NAC = 1	NAC = 24	NAC = 11.4	NAC = 43
		SF = 97	SF = 77	SF = 56	SF = 58	SF = 29	SF = 26	SF = 1	SF = 15.9	SF = 17.3	SF = 72
Rovers (2021)	NR	–	–	–	–	–	–	NAC = 0	NAC = 22	–	–
								SF = 0	SF = 33		
Beal (2020)	18.6^1^	NAC = 81*	NAC = 44*	NAC = 38*	NAC = 52*	NAC = 28*	NAC = 20*	NAC = 2	NAC = 22	NAC = 13.8	NAC = 32.7
		SF = 76	SF = 33	SF = 18	SF = 56	SF = 20	SF = 11	SF = 3	SF = 17	SF = 13	SF = 22
Leimkuhler (2019)	NR	–	–	–	–	–	–	NAC = 0	Not graded	–	–
								SF = 0			
Van Eden (2017)	29.8	NAC = 94*	NAC = 50*	NAC = 32*	NAC = 76*	NAC = 25*	NAC = 21*	NAC = 2.6	NAC = 26.9	NAC = 19.5*	NAC = 36.9
		SF = 90	SF = 44	SF = 15	SF = 74	SF = 18	SF = 17	SF = 2.4	SF = 29	SF = 16	SF = 34
Devilee (2016)	30.8	NAC = 100*	NAC = 89	NAC = 71*	–	–	–	NAC = 0	NAC = 24	–	NAC = NR
		SF = 89	SF = 50	SF = 23				SF = 1.5	SF = 16.7		SF = 38.6
Baratti (2014)	44.9	–	–	NAC = 37.2	–	–	–	3 (Entire group)	24 (Entire group)	–	–
				SF = 48.1							
Ceelen (2014)	18	NAC + BEV = 96*	NAC + BEV = 72*	NAC + BEV = NR*	–	–	–	2.4 (Entire group)	35 (Entire group)	–	NAC+BEV = 39
		NA C = 75	NA C = 30	NAC = 12.5							NAC = 22
		SF = 75	SF = 39	SF = 24							SF = 25
Passot (2012)	36.2	NAC = 79*	NAC = 53*	NAC = 41*	–	–	–	3.8 (Entire group)	21.8 (Entire group)	–	NAC = 36*
		SF = 67	SF = 39	SF = 23							SF = 23
Elias (2010)	45	–	NAC = 40.5	NAC = 27	–	–	–	3.3 (Entire group)	31 (Entire group)	–	NAC = 30
			SF = 42	SF = 26							SF = 30
Glehen (2004)	53	–	–	–	–	–	–	4 (Entire group)	22.9 (Entire group)	–	NAC = 19.2
											SF = 20.4

*DFS* disease-free survival; *OS* overall survival; *NAC* neoadjuvant chemotherapy; *SF* surgery first; *BEV* bevacizumab; *NR* not reached

*Estimated based on survival curve

### Mortality

**Fig. 2:**
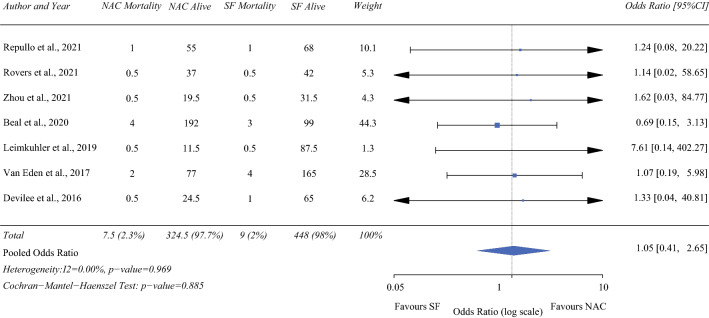
Forrest plot demonstrating perioperative mortality risk associated with neoadjuvant chemotherapy compared to surgery first

Seven of the studies (425 NAC/568 SF)^12,13,16,19,21–23^ included in the meta-analysis reported on perioperative mortality. This ranged from 0 to 2.6% in the NAC group, and from 0 to 3% in the SF group. Three studies reported 30-day mortality and four did not specify a timescale. On pooled analysis, patients who received NAC had no associated increased mortality (hazard ratio [HR] 1.05; 95% confidence interval [CI] 0.41-2.65, *P* = 0.885), with zero heterogeneity (I^2^ = 0%; Fig. 2).

### Morbidity

**Fig. 3:**
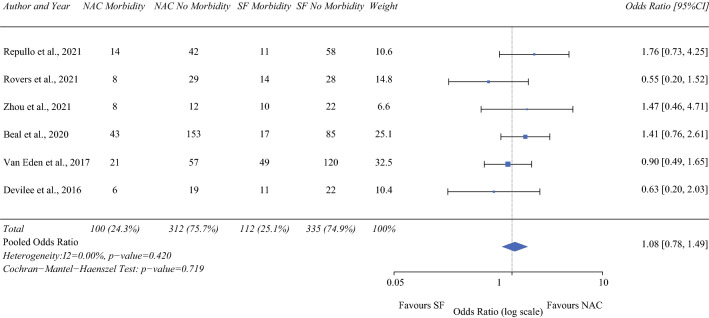
Forrest plot demonstrating the risk of perioperative major morbidity with neoadjuvant chemotherapy compared to surgery first

Six studies (413 NAC/480 SF)^12,13,16,19,21,23^ reported on major (grade III/IV) perioperative morbidity, which ranged from 22% to 40% in the NAC group and from 16.7% to 33% in the SF group. Four studies reported on major morbidity in accordance with the Clavien-Dindo classification system,^13,16,19,23^ whereas two reported using Common Terminology Criteria for Adverse Events (CTCAE).^12,21^ On pooled analysis, there was no difference in grade III/IV morbidity between the two groups (HR 1.08; 95% CI 0.78-1.49; *P* = 0.719), with zero heterogeneity (I^2^ = 0%; Fig. 3).

### Overall Survival (3-year)

**Fig. 4:**
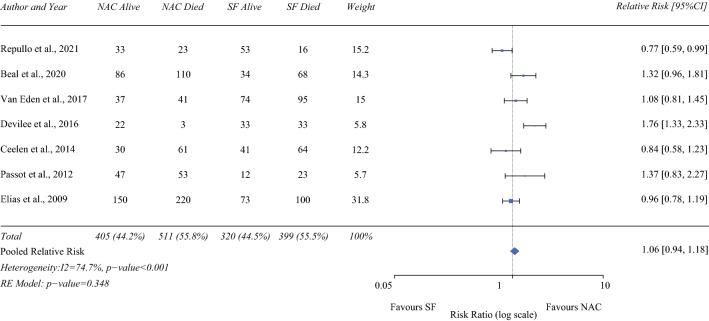
Forrest plot demonstrating the meta-analysis of 3-year overall survival

Seven studies (916 NAC/719 SF)^13,16–18,21,23,24^ reported on 3-year overall survival. On pooled analysis, NAC patients experienced no significant improvement in 3-year survival compared with SF patients (RR 1.06; 95% CI 0.94-1.18; *P* = 0.348), with high heterogeneity (I^2^ = 74.7%; Fig. 4).

### Overall Survival (5-year)

**Fig. 5:**
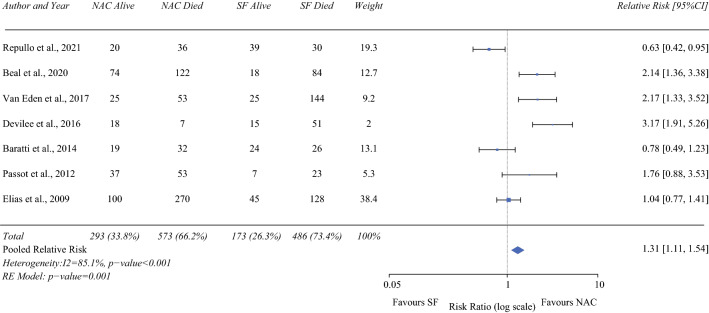
Forrest plot demonstrating the meta-analysis of 5-year overall survival

Seven studies (866 NAC/659 SF)^3,13,16,18,21,23,24^ reported on 5-year overall survival. On pooled analysis NAC patients had a significantly better 5-year overall survival than SF patients (RR 1.31; 95% CI 1.11–1.54; *P* < 0.001). Heterogeneity was high (I^2^ = 85.1%; Fig. 5). Given the study by Devilee et al.^13^ had such a stark difference in survival between groups at 5 years (NAC: 71%, SF: 23%), the pooled analysis was redone without these data. This resulted in a persistent pooled survival advantage for NAC patients (RR 1.22; 95% CI 1.03–1.45; *P* = 0.024).

### Disease-free Survival (1-year)

**Fig. 6:**
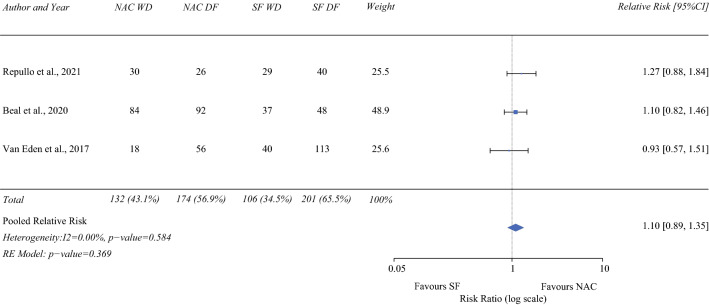
Forrest plot demonstrating the meta-analysis of 1-year disease-free survival

Disease recurrence was determined by computed tomography ± positron emission tomography in one study,^23^ by “clinical or radiographic” evidence in another,^16^ and by unknown means in the third.^21^ Three studies (306 NAC/307 SF)^16,21,23^ reported on 1-year disease-free survival. On pooled analysis, NAC patients received no disease-free survival advantage at 1 year compared with SF patients (RR 1.10; 95% CI 0.89–1.35; *P* = 0.369), with low heterogeneity (I^2^ = 0%; Fig. 6).

### Disease-free Survival (3-year)

**Fig. 7:**
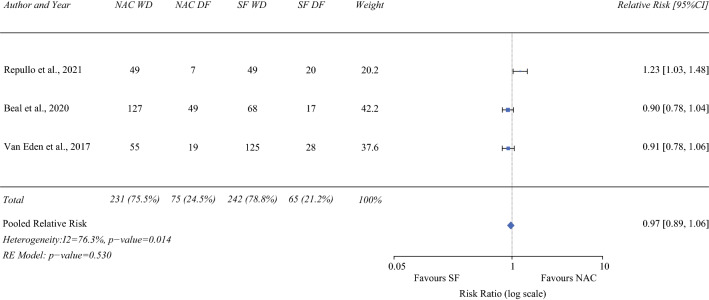
Forrest plot demonstrating the meta-analysis of 3-year disease-free survival

Three studies (306 NAC/307 SF)^16,21,23^ reported on 3-year disease-free survival. On pooled analysis, there was no difference in 3-year, disease-free survival between the groups (RR 0.97; 95% CI 0.89–1.06; *P* = 0.53), with high heterogeneity (I^2^ = 76.3%; Fig. 7).

## Discussion

This meta-analysis of 12 studies (*n* = 2,463 patients) demonstrates no significant increase in perioperative major morbidity and mortality in selected patients with CRPM who received preoperative chemotherapy followed by cytoreductive surgery and HIPEC. Despite the predominance of retrospective, low-quality nature of the interpreted data, statistical heterogeneity for both of these outcomes was zero, which allows for relatively confident interpretation. Furthermore, it is worth noting that 41% of patients received bevacizumab as part of their neoadjuvant chemotherapy regimen, further dispelling concerns of its antiangiogenic effects on wound healing and anastomotic integrity^25^ when ceased appropriately in the preoperative setting. However, interpretation of a survival benefit with NAC is less convincing. No benefit was evident in 1-year and 3-year DFS, although these outcomes were infrequently reported upon across the meta-analysed studies. Examining OS, there was similarly no difference in 3-year survival; however, at 5 years, patients who received NAC had a significant survival advantage.

Supporting rationale for NAC use in patients with “high” PCI/unresectable disease are numerous. The potential of tumour downsizing and elimination of micrometastatic disease increases the likelihood of patients reaching potentially curable surgery, but assessment of factors, such as tumour biology and chemosensitivity, should be considered essential elements to appropriate patient selection. The improvement of 5-year OS in patients with NAC shown in this study may be because of many of these discussion points. The question remains as to whether these aspects hold true for patients with isolated, resectable CRPM.

On subgroup analysis, the PRODIGE 7 trial showed no association between preoperative chemotherapy and overall survival, although interestingly, NAC was deemed a positive prognostic indicator for disease-free survival in the CRS & HIPEC group (HR 0.45; 95% CI 0.25–0.84; *P* = 0.04). However, data are lacking on the outcomes of patients who were not offered CRS and HIPEC post NAC. It is thus possible that the perceived survival advantage is attributable to patient selection rather than to the chemotherapy itself. The exact benefit, if any, of preoperative systemic chemotherapy may not be decided upon until a prospective, randomised trial data is published. Indeed, in the only randomised trial included in this meta-analysis, Rovers et al., demonstrated the feasibility, safety, and ability of NAC to induce a pathological response.^19^ This acts as a prelude to the much-awaited survival data that the subsequent phase III trial, CAIRO6, will provide.^10^

Although not eligible for inclusion here, growing evidence is building on the pathological response of CRPM to NAC. Complementing the complete pathological response (pCR) of 24% shown in the phase II trial by Rovers et al. ^19^ rates of 11–28% have been described elsewhere,^26–28^ with an accompanying favourable prognosis as is well documented with pCR post neoadjuvant chemoradiotherapy in primary rectal cancer.^29^ In a prospective study comparing 120 patients with and without a pCR, Bhatt and colleagues^26^ reported that 80% of patients with a surgical PCI of ≤3 exhibited a pCR, raising further questions as to the benefit of HIPEC in low PCI subgroups. The potential inclination of a surgeon might be to offer CRS & HIPEC upfront to patients with a “low” PCI, but if NAC can induce a pCR without compromising perioperative outcomes, then potentially these patients should be offered initial systemic chemotherapy followed by CRS alone. Interestingly, PRODIGE 7 showed no benefit in HIPEC in patients with a PCI <11 in a heavily pretreated population (83% receiving NAC). Again, this apparent lack of benefit in adding HIPEC may be due to a high pCR rate in this patient subgroup.

The impact of adjuvant chemotherapy was not addressed in this study. It is worth noting, however, that 46% and 58% of the NAC and SF groups (where reported), respectively, received postoperative chemotherapy. The influence of adjuvant systemic chemotherapy on untreated resected CRPM populations has been assessed, albeit retrospectively. A population-based cohort study of almost 400 patients from the Netherlands showed survival benefit in adjuvant chemotherapy (39 months median OS) versus active surveillance (25 months median OS) in patients receiving upfront CRS and HIPEC.^30^ Similarly from Sweden, a recent retrospective study of 131 consecutive patients reported a median OS of 40 months after complete cytoreduction, with 60% of the study population receiving adjuvant chemotherapy.^31^ The authors question the need for NAC due to the favourable reported survival. Perhaps, for the purposes of survival, the timing of systemic chemotherapy is inconsequential. However, given the relatively high major morbidity associated with CRS and HIPEC (15–30%)^32^ and the effect this may have on patients’ ability to receive adjuvant chemotherapy, one could postulate the benefit of giving the total desired amount of systemic chemotherapy in a neoadjuvant fashion, following in the footsteps of total neoadjuvant therapy (TNT) in rectal cancer.^33^

The largely retrospective design of the included studies leads to an expected high level of selection bias. From experiences at our own centre, further selection bias may lie in those offered upfront surgery. Patients who relapse with resectable peritoneal disease shortly after or even during adjuvant systemic chemotherapy for their index surgery, frequently undergo CRS and HIPEC upfront. Not only have these patients already displayed an aggressive tumour phenotype but also early chemo resistance, possibly precluding any long-term survival.

The lack of reported data on subsequent chemotherapy/surgical intervention (for example redo CRS & HIPEC), which may prolong survival, adds further complexity to the interpretation of the impact of NAC from these studies. This limitation weakens the selection of overall survival as an endpoint in this study.

It is uncertain as to why an overall survival benefit in the neoadjuvant therapy group became significant at 5 years but was not at 3 years. Although not significant at 3 years, the pooled analysis did slightly favour neoadjuvant chemotherapy (RR 1.06). Nevertheless, this discrepancy raises debate as to whether subsequent treatment played a role in survival, even though it appeared that more surgery-first patients received adjuvant systemic chemotherapy, although data on type and number of cycles received was not available. Although one of the studies^17^ included in the 3-year analysis did not reach adequate follow-up to be included at 5 years, it is unlikely that the 5-year relative risk would change given the trajectory of the Kaplan-Meier curves of each respective subgroup in that study.

Indications for administering systemic chemotherapy rather than upfront surgery were not clear and data surrounding patients not offered surgery post NAC was not reported. Furthermore, diverse differences in both systemic and intraperitoneal chemotherapy regimens (many of which are not widely utilised) add to the heterogeneity of a highly complex and multifaceted disease process.

## Conclusions

This systematic review and meta-analysis suggests that neoadjuvant chemotherapy is feasible, and when administered to patients who proceeded to surgery, did not have any direct ill effects on postoperative complications. An overall survival advantage at 5 years was shown in patients who received NAC followed by surgery; however, significant limitations temper confidence in interpretation of this. As we wait for randomised data to provide some clarity on this controversial topic, national and international guidance will continue to be based on low-level evidence.

## Supplementary Information

Below is the link to the electronic supplementary material.

Supplementary file1 (DOCX 11 kb)
