# Supplementary material for: The Impact of Neoadjuvant Chemotherapy on the Surgical Management of Colorectal Peritoneal Metastases: A Systematic Review and Meta-Analysis
Source: Ann Surg Oncol. 2022 Apr 9;29(11):6619–31. doi: 10.1245/s10434-022-11699-7 (PMC9492604; doi:10.1245/s10434-022-11699-7)
Supplement: Supplementary file 1 — Supplementary file1 (DOCX 11 kb) [file 10434_2022_11699_MOESM1_ESM.docx]

**Supplementary Data 1.** Search Strategy

1. (colorectal cance* or colorectal neoplas* or colorectal tumo* or colorectal carcinom*).mp. [mp=title, abstract, original title, name of substance word, subject heading word, floating sub-heading word, keyword heading word, organism supplementary concept word, protocol supplementary concept word, rare disease supplementary concept word, unique identifier, synonyms]

2. (CRS or cytoreductive surger*).mp. [mp=title, abstract, original title, name of substance word, subject heading word, floating sub-heading word, keyword heading word, organism supplementary concept word, protocol supplementary concept word, rare disease supplementary concept word, unique identifier, synonyms]

3. (HIPEC or hyperthermic intraperitoneal chemotherap* or intraperitoneal chemohyperthermia or IPCH).mp. [mp=title, abstract, original title, name of substance word, subject heading word, floating sub-heading word, keyword heading word, organism supplementary concept word, protocol supplementary concept word, rare disease supplementary concept word, unique identifier, synonyms]

4. (peritoneal or peritoneum).mp. [mp=title, abstract, original title, name of substance word, subject heading word, floating sub-heading word, keyword heading word, organism supplementary concept word, protocol supplementary concept word, rare disease supplementary concept word, unique identifier, synonyms]

5. (systemic chemotherapy or neoadjuvant chemotherapy or adjuvant chemotherapy or perioperative chemotherapy).mp. [mp=title, abstract, original title, name of substance word, subject heading word, floating sub-heading word, keyword heading word, organism supplementary concept word, protocol supplementary concept word, rare disease supplementary concept word, unique identifier, synonyms]

6. 1 and 2 and 3 and 4 and 5
